# MicroRNAs in neuroblastoma tumorigenesis, therapy resistance, and disease evolution

**DOI:** 10.20517/cdr.2019.68

**Published:** 2019-12-19

**Authors:** Natarajan Aravindan, Karthikeyan Subramanian, Dinesh Babu Somasundaram, Terence S. Herman, Sheeja Aravindan

**Affiliations:** 1 Department of Radiation Oncology, University of Oklahoma Health Sciences Center, Oklahoma City, OK 73104, USA.; 2 Stephenson Cancer Center, Oklahoma City, OK 73104, USA.

**Keywords:** miRNAs, progressive neuroblastoma, therapy resistance, oncomiRs, tumor suppressor miRs, tumor progression

## Abstract

Neuroblastoma (NB) deriving from neural crest cells is the most common extra-cranial solid cancer at infancy. NB originates within the peripheral sympathetic ganglia in adrenal medulla and along the midline of the body. Clinically, NB exhibits significant heterogeneity stretching from spontaneous regression to rapid progression to therapy resistance. MicroRNAs (miRNAs, miRs) are small (19–22 nt in length) non-coding RNAs that regulate human gene expression at the post-transcriptional level and are known to regulate cellular signaling, growth, differentiation, death, stemness, and maintenance. Consequently, the function of miRs in tumorigenesis, progression and resistance is of utmost importance for the understanding of dysfunctional cellular pathways that lead to disease evolution, therapy resistance, and poor clinical outcomes. Over the last two decades, much attention has been devoted to understanding the functional roles of miRs in NB biology. This review focuses on highlighting the important implications of miRs within the context of NB disease progression, particularly miRs’ influences on NB disease evolution and therapy resistance. In this review, we discuss the functions of both the “oncomiRs” and “tumor suppressor miRs” in NB progression/therapy resistance. These are the critical components to be considered during the development of novel miR-based therapeutic strategies to counter therapy resistance.

## INTRODUCTION

Neuroblastoma (NB) is the prime extracranial solid tumor in infants^[1,2]^. It is a tremendously lethal malignancy that accounts for ~10% of all pediatric cancer deaths^[3–5]^. With 50% of the diagnosed cases designated as high-risk progressive disease, the overall survival (OS) rates remain around 40%, despite intensive multimodal clinical therapy (IMCT)^[6]^. Given the disease’s heterogeneity, resistance, and poor hematological reserve, cure for high-risk disease is rare, with < 10% 5-year OS and 2% 10-year survival (*vs.* 38%–71% for low-risk disease)^[7,8]^. NB is characterized by hematogenous metastasis^[9]^. Since NB is a highly heterogeneous disease and there is a continuous acquisition of genetic and molecular rearrangements in such heterogeneous clones that contribute to the therapy resistance, tumor relapse, and disease evolution, we must understand the acquired/adapted reorganizations if we are to successfully treat NB. Although epigenetic, genetic, and molecular rearrangements have been causally linked to disease evolution beyond IMCT, in this review we directed our focus on the role of microRNAs (miRs), particularly those involved in tumor dissemination and progression, generally designated as “metastamiRs”.

NB is derived from neural crest cells (NCCs) of sympathoadrenal lineage (SAPs)^[6,10–13]^, when these cells cease to differentiate^[14]^. These SAPs undergo epithelial-to-mesenchymal transition (EMT), augmenting NCCs’ migratory abilities and with a complex interplay of sequential molecular events orchestrating functional response (e.g., catecholamine biosynthesis)^[15]^ that dictates NB genesis. Since NB genesis could be drawn from SAPs destined for various lineages^[16]^, NB is generally characterized with biological and clinical heterogeneity^[17–19]^. In the last two decades, significant advances have been made in staging, risk stratification, and treatment strategies. Briefly, for clinically stable stage I/II disease, surgery in which the tumor mass is excised is the prime treatment strategy, without any chemotherapy or other treatments^[20]^. However, for high-risk disease (stages III/IV), IMCT comprises the induction phase, with alternating regimens of high-dose chemotherapeutic drugs and load reduction surgery; followed by the consolidation phase with more intensive chemotherapy, along with radiotherapy and stem cell transplant; and finally the maintenance phase with retinoid drug treatment, immunotherapy, and immune-activating cytokine treatment. Despite IMCT, the disease evolves with frequent relapses in decreasing time intervals^[21,22]^, reflecting the continuous acquisition of genetic and molecular rearrangements in the undifferentiated/poorly differentiated NB cells^[23–25]^, and warrants a better understanding of the underlying genetic/molecular mechanisms. Defining such events would allow us to develop improved targeted therapeutic strategies for better control of the disease evolution and, subsequently, to achieve the desired clinical outcomes.

Discovered in 1993^[26]^, miRs are a group of small non-coding RNAs of approximately 22 nucleotides in length that regulate gene expression at the post-transcriptional level. Thus far, a total of 1917 miRs have been identified in humans (CRCh38, miR Base). Studies have documented cell-/tissue-specific expression of select sets of miRs with roles in shaping cellular identity and cell-specific function^[27,28]^. miRs exert multidimensional control on gene expression, through direct DNA interaction, translational repression, or by routing mRNA degradation^[29]^. In general, miRs play crucial roles in maintaining homeostasis in cellular systems. Rearrangements in miR profiles could derail balance in development, differentiation, and growth, leading to diseases such as cancer. A plethora of studies have documented the functional role of miRs in regulating oncogenes, tumor suppressors, and genes involved in the cell cycle, apoptosis, cell migration, and angiogenesis^[30–37]^. Changes in miR expression associated with cancer development can occur through a manifold of mechanisms, each of which is a critical event that could greatly alter early development and give rise to the pathogenesis of childhood cancers. In this regard, researchers have indicated the benefit of non-invasive screening for serum miRs in the diagnosis and risk stratification of childhood solid tumors^[38]^.

Functionally, the miR serves as a guide to the target mRNA through base-pairing and thereby negatively regulates the target expression. The complementarity level between miR and mRNA determines the mechanism of silencing, whether through cleavage of target mRNA with subsequent degradation or through translation inhibition. The biogenesis of miRs, mechanisms of action, and their roles in cancer were reviewed in detail elsewhere^[39]^. With about 60% of human protein-coding genes controlled by miRs, independent studies have consistently demonstrated the role of miRs in regulating cellular functions, including the cell cycle, proliferation, differentiation, apoptosis, and metabolism. Aberrant alterations (expression/suppression) in miRs have been shown to prompt carcinogenesis, tumor progression, cancer evolution, cancer stem cells, autophagy, multi-drug resistance (MDR), EMT, migration, invasion, metastasis, and others^[40–42]^. miRs can be designated as oncomiRs (inhibit tumor suppressor genes), metastamiRs (inhibit tumor stabilization genes), or tumor suppressor miRs (annul oncogenes), based on the targets regulated^[43,44]^. Studies have also shown tumor- (*vs.* normal tissue), tumor type-, and disease type- (primary or metastatic) specific miR signatures^[45–47]^. For instance, reduced levels of miR-Let-7 (targets proto-oncogene RAS)^[48]^ and overexpression of polycistronic miR cluster miR-17-92^[30,31]^ were shown in lung cancer, while miR-15 and miR-16 are frequently deleted in chronic lymphocytic leukemias^[49]^. However, attaining the cancer type-specific miRs blueprint is still in the early stages for various reasons, including continuously evolving signatures with disease progression, therapy resistance, cancer site, and others; exclusion of prognosis-relevant miRs with putative oncogenic functions based on their silent state or low expression; and inclusion of miRs that are capable of regulating targets of both arms (promotion and inhibition) of signaling where balance might shift with disease evolution. Furthermore, beyond the gene negative regulator function of miRs, studies have also indicated that miRs can moderate transcription^[50,51]^ or activate translation^[52]^.

## ROLE OF MIRS IN NB GENESIS

**Figure 1.**
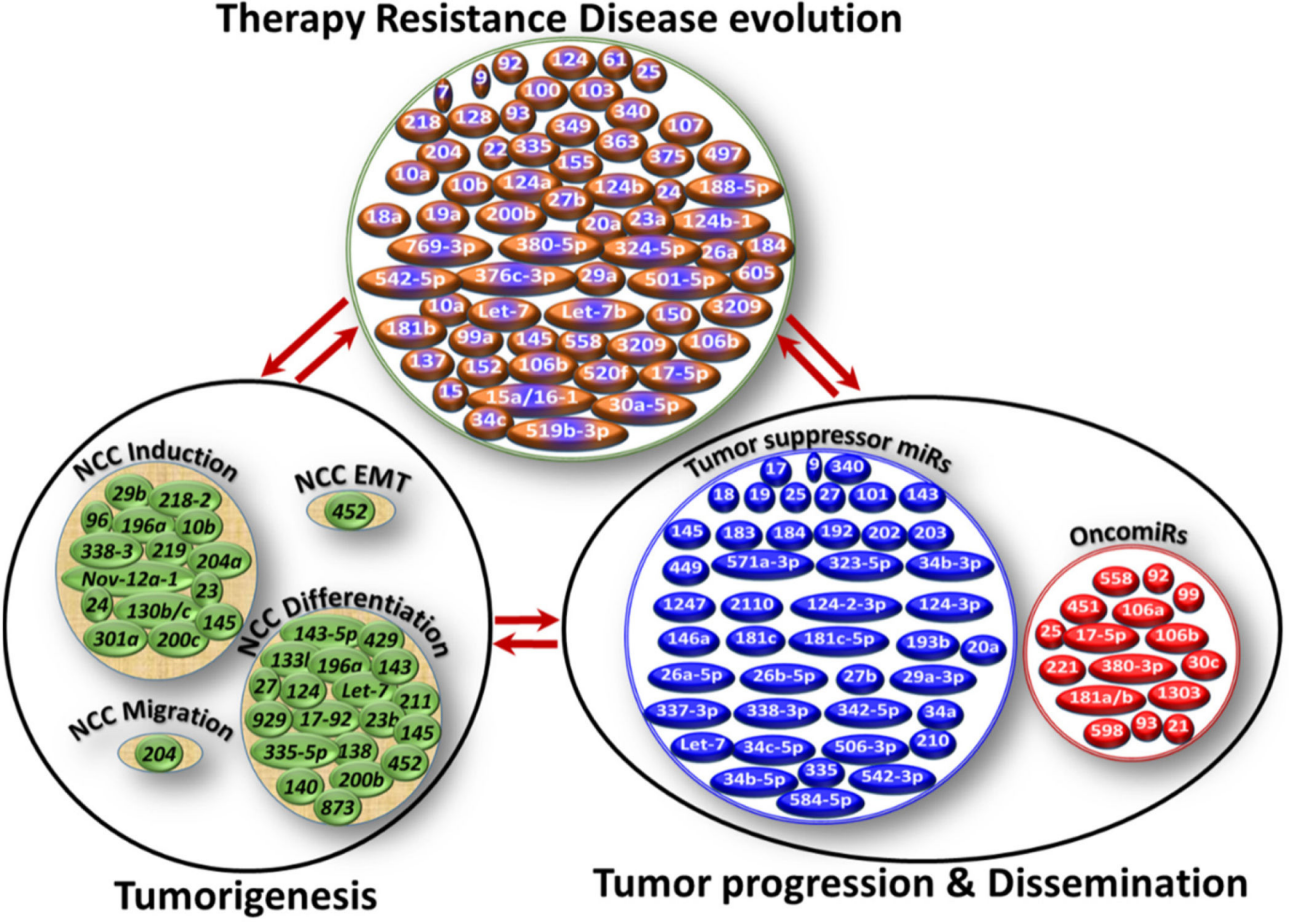
Schematic representation of miRs involved in neuroblastoma (NB) genesis, tumor progression, and therapy resistance. While cellular function- and NB process-specific miR modulation were realized, it was also clear that the functional roles of miRs (e.g., miR-9, miR-92) are conserved across the process, at least in the NB setting. In-depth analysis and crisscross comparisons of the documented functional roles of miRs across NB biological processes showed NB process (genesis, progression, therapy resistance) - specific miR(s) regulation/deregulation; within a process, function-specific miR(s) modulation; activation of function-specific miRs is complemented with compromised incompatible miR(s) (e.g., oncomiR activation with concomitant decrease of tumor suppressor miR or vice versa) and; the involvement of select miRs across the function within a process and also across the processes of NB evolution

Researchers have shown that the tissues of early neural crest development are rich in miR diversity, and that select sets of miRs are explicitly expressed in NCCs^[53]^, the primordial source from which NB originates. During development, miRs fine-tune protein levels, which contribute to the progressive changes in gene expression profiles of different cell types and/or maintain steady-state levels of genes for programmed processes, including NCCs induction, differentiation, delamination, and migration [Figure 1]^[54]^. Furthermore, miRs have been shown to regulate EMT, the critical step leading from SAPs to NB genesis, in NCCs, dysregulation of which could contribute to neural crest disorders^[55]^. More importantly, the functional role of miRs in NB genesis is clearly documented in studies that showed preferential downregulation of miRs in an N-MYC-driven NB model appropriating the gene signature for tumor genesis, as well as to sustain N-MYC expression^[56]^. A recent miR-mRNA interactions study for network modeling in NB tumorigenesis designated miR-204 as a tumor suppressor. Expression of miR-204 directly bound with N-MYC mRNA, repressed its expression, and inhibited NB tumorigenesis^[57]^. Mostly, the function of miRs in NB has been indicated through high-content screening strategies coupled with sequential candidate function-finding strategy (using miR mimics or inhibitors) in terms of cell differentiation^[58–60]^. Du and colleagues documented the differentiation-inducing and oncosuppressive function of miR-2110, one among many neurite-inducing miRs identified by high-content screening^[58]^, through targeted inhibition of *TSKU*^[61]^.

## ROLE OF MIRS IN NB DISEASE PROGRESSION AND METASTASIS

Deregulation of miRs may be an important mechanism that contributes to pathogenesis and heterogeneity of NB^[62,63]^. Clinical behaviors of NB may be considerably correlated with their specific genetic abnormalities^[64]^ (e.g., amplifications, deletions, point mutations) and relatively rapid epigenetic changes (e.g., DNA methylations, histone modifications)^[65]^. For instance, studies have documented a strong inverse association between miR-2110 (low tumor levels) and its target, *TSKU* (high tumor mRNA levels), and further demonstrated a significant correlation of both low miR-2110 and high *TSKU* mRNA with poor patient survival^[61]^. Likewise, Chen and Stallings^[66]^ showed underexpression of a specific subset of miRs in N-MYC-amplified NB cells, and targeting N-MYC restored these crucial miRs that play functional roles (e.g., miR-184 in inducing apoptosis). This data suggests that N-MYC could induce NB pathogenesis by regulating miRs that promote NB cell differentiation, apoptosis, and others.

Deregulation of miRs in malignant NB could be due to N-Myc amplifications, chromosomal deletion, or abnormal epigenetic regulation^[62,67]^. N-Myc binds to the promoter region of an array of miRs and regulates their expression. An independent study showed that at least 37 miRs were differentially expressed in NB with N-Myc amplification compared with N-Myc non-amplified NB^[68]^. While several oncomiRs (miR-17-5p, miR-92, miR-93, miR-99, miR-106a, and miR-221) are upregulated with N-Myc amplification in progressive NB, suppression of tumor suppressors like miR-34a is also observed with N-Myc amplification. In addition, N-Myc activates methyl-transferases and prompts methylation of target genes. To that note, deregulated epigenetic machinery can aberrantly modify the promoters of miRs in NB. Researchers have identified a panel of oncomiRs and tumor suppressors (let-7, miR-101, miR-202, miR-9, miR-34a, miR-340, miR-184, and miR-335) that are controlled under epigenetic regulation (aberrant DNA methylation or histone modification) in malignant NB^[69,70]^. For instance, Banelli and colleagues identified that miR-34b-3p, miR-34b-5p, miR-34c-5p, and miR-124-2-3p were significantly hypermethylated and consequently downregulated, particularly in the subset of NB patients at high risk of progression^[71]^. Conversely, miR-221 has been shown to increase the expression of N-MYC by directly targeting Nemo-like Kinase (NLK), regulating the cell cycle, and promoting the growth of NB cells^[72]^.

Since evolving NB harbors a characteristic miR profile and given miRs’ biomarker designation for NB diagnosis, therapy response, and prognosis^[62]^, stabilizing altered miR species could improve therapeutic strategies and yield a better outcome. An array of miRs functions as tumor suppressors as well as tumor evolution stabilization candidates, deregulation of which heavily contributes to NB progression [Table 1]. For example, stabilizing miR-203 directly targeted Sam68 translation and thereby inhibited NB cell proliferation, migration, and invasion^[73]^. Likewise, reinforcing miR-337-3p and miR-584-5p has been indicated to inhibit NB cell growth, invasion, metastasis, and angiogenesis by targeting MMP14^[74,75]^. In this line of tumor suppressor miRs, miR-449a has been extensively investigated for its beneficial functions in various tumors, including lung, liver, pancreatic, breast, bone, brain, prostate, head and neck, ovarian, endometrial, and bladder cancer^[76–81]^. In the context of NB, miR-449a has been shown to inhibit cell survival and growth through two converging mechanisms: inducing cell differentiation and cell cycle arrest^[59]^.

While miR-449a dictates NB cell differentiation by selectively targeting MFAP4, PKP4, and TSEN15, in parallel it also affects cell cycle arrest through downregulation of CDK6 and LEF1^[59]^. Previously, utilizing a high-content morphological screening approach, the same group identified three miR seed families (seed region is 5’ end of the mature miRNA consisting of similar eight nucleotides that are involved in inducing NB cell differentiation^[58]^. In this study, they showed that miR-506-3p and miR-124-3p have unparalleled differentiation-inducing capability and they exert their tumor-suppressive function in part by downregulating their targets, CDK4 and STAT3. miR-124 is most specifically expressed in the nervous system, plays a prominent role in neuronal differentiation, and is increased during neural development^[82,83]^. They also recognized that miR-506-3p is dramatically upregulated in differentiated NB cells, indicating its crucial role in differentiation and tumorigenesis. Similar to miR-449a, miR-193b is a well-documented and designated tumor suppressor in various human cancers^[84–88]^. miR-193b showed significantly lower expression in NB and corresponded to increased cell viability and proliferation^[89]^. Functionally, miR-193b induces G1 cell cycle arrest and NB cell death by selectively inhibiting the expression of N-MYC, Cyclin D1, and MCL1.

In parallel, an independent study identified the NB tumor suppressor function of miR-145, a miR that is enriched in germ-line and mesoderm-derived tissues^[90]^. Since its first record of downregulation and its role in the tumorigenesis of early stage colorectal cancer, its tumor suppressor function has been widely recognized in many human cancers^[91–96]^, including nervous system tumors such as glioblastoma and CNS lymphoma^[97]^. Functionally, in NB, it has been shown that miR-145 targets HIF-2α and inhibits the hallmark physiognomies (growth, invasion, metastasis, and angiogenesis) of tumor progression and metastasis^[90]^. Another study indicated that miR-27b directly targets the 3’UTR of PPARγ and regulates NFκB, NHE1, and tumor growth and progression^[98]^. miR-542-3p and 5p, the miRs that inversely associate with poor prognosis in NB, directly target survivin and dictate tumor-suppressive functions^[99]^.

Studies assessing the tumor suppressor functions of miRs in NB setting have shown that miR-34a and miR-184 are significantly underexpressed in NB^[100,101]^. Functionally, miR-34a targets N-MYC^[100]^, while miR-184 targets AKT and orchestrates their tumor-suppressive function^[102]^, miR-34a induces cell cycle arrest and apoptosis activation in NB cells^[103]^. Furthermore, it has been shown that CDK1 regulates miR-34a in NB cells^[104]^. Inhibition of CDK1 dictates increased expression of miR-34a, which in turn downregulates N-MYC and mediates the Survivin loss associated with cell death. Comparing the metastatic NB tissues, many studies identified the deregulation of miRs^[105]^, however, only a few in-depth reports are available on the mechanism of regulation and the function of miRs in disease evolution. Studies that attempted to define the mechanism(s) of action (cell death, inhibition of invasion, metastasis) of miRs in disease evolution identified: miR-335, which targets many gene targets in the non-canonical TGFβ signaling pathway^[106]^; miR-9, which targets MMP14^[107]^; miR-210, which targets BCl2^[108]^; and miR-181c, which targets Smad7^[109]^.

Consistently, it has been shown that miR-181c was significantly downregulated in metastatic tissues compared with primary NB tissues^[109]^. Likewise, Purello and colleagues showed that downregulation of miR-29a-3p, miR-34b-3p, miR-181c-5p, and miR-517a-3p in NB tissues (compared with normal adrenal gland) was primarily due to the hypermethylation of their promoters^[110]^. Since these tumor suppressors are disallowed in NB, uncontrolled expression of their targets CDK6, DNMT3a, DNMT3B, CCNA2, and E2F3 dictates disease progression. Deubzer and colleagues reported that transcription of miR-183 is significantly repressed by N-MYC and is highly induced in N-MYC-amplified NB in response to pan-HDAC inhibitor and cyclic tetrapeptide^[111]^. miR 183 expression studies by this group later identified 85 targets, including all 6 members of the minichromosome maintenance complex (MCM2, MCM3, MCM4, MCM5, MCM6, MCM7) that is critical for initiation and elongation during DNA replication^[112]^.

The transcriptional regulator LMO1 has been shown to promote NB cell proliferation and has been designated as an NB susceptibility gene by GWAS^[113]^. Later, this group identified that LMO1 indirectly downregulates at least 18 miRs, including seven miRs of the Let7 family (7a-5p, 7b-5p, 7c, 7e-5p, 7f-5p, 7g-5p,7i-5p)^[114]^. The proliferation inhibition function of these seven members of the Let7 family was extensively documented. The regulation of these candidates by LMO1 in NB indicates well-orchestrated, cluster-focused control of tumor suppressor miRs in disease evolution. A number of mechanisms that could deregulate the Let-7 family in NB has been reported and was recently reviewed in detail by Daley and colleagues^[115]^.

Many studies have identified and characterized tumor-suppressive miRs in NB and defined their mechanistic signaling flow-through in orchestrating tumor progression and disease resistance. A very recent observation with functional high-throughput screening in both *in vitro* and *in vivo* models identified the NB tumor-suppressive function of miR-323-5p and miR-342-5p, and indicated that the effect occurs through directly targeting CCND1, CHAF1A, INCENP, and Bcl-Xl^[116]^. Interestingly, two tumor suppressor miRs, miR-26a-5p and miR-26b-5p, which directly regulate LIN28B, are in turn affected by N-MYC expression, leading to N-MYC-driven LIN28B-mediated oncogenic processes^[117]^. Chen and colleagues identified PREX2a as a direct target of miR-338-3p^[118]^. PREX2a can directly interact with PTEN to inactivate its lipid phosphatase activity, leading to accumulation of PIP3 and, as a consequence, increased AKT phosphorylation, which in turn promotes NB cell survival, cell cycle progression, and tumor growth. miR-338-3p-dependent inhibition of PREX2a affects PTEN/AKT pathway-dependent tumor progression. miR-338-3p is transcribed from the intron 8 of the apoptosis-associated tyrosine kinase (*AATK*) gene, located on chromosome 17q25, playing a critical role in promoting cell death, neuronal differentiation, and neurite extension^[119,120]^. Assessing the biological function of miR-1247 in NB, it was realized that ZNF346 is the direct target of miR-1247 and its targeted inhibition of ZNF346 suppresses cell proliferation and induces cell cycle arrest and NB cell death^[121]^. Similarly, miR-146a has been shown to inhibit NB cell growth and promote apoptosis by directly down-modulating its target, BCL11A^[122]^. Similarly, miR-192 has been reported as an independent prognostic marker for relapse in NB patients; miR-192 directly targets Dicer1 and was functionally designated as a tumor suppressor miR^[123]^.

Understanding the deregulation of designated oncomiRs in NB cells and recognizing their driving role in disease progression, dissemination, and clinical outcomes is of great importance [Table 1]. For instance, oncomiR-558 was shown to directly facilitate the transactivation and translation of HPSE and its downstream target VEGF, and contribute to NB tumor progression^[124]^. Studies have also demonstrated that miR-558 directly facilitates Ago2- and eIF4E-dependent HIF2α expression and dictates NB growth, invasion, metastasis, and angiogenesis^[125]^. Likewise, lower levels of miR-451 were inversely correlated with NB growth, invasion, and migration. It has been shown that miR-451 directly inhibits macrophage inhibitory factor, which critically mediates the biological effect of miR-451 in NB^[126]^. Stem-loop sequence miR-1303, on the other hand, has been shown to promote NB cell proliferation by selectively targeting GSK3β and SFRP1^[127]^. While GSK3β is critical for the phosphorylation of Cyclin D1, SFRP1 directly binds with WNTs and inhibits the WNT/β-catenin pathway, indicating that miR-1303-directed inhibition of these candidates could endorse disease progression. NB patients with high levels of SFRP1 have shown good prognosis.

In the highly tumorigenic sub-population of CD114^+^ cells, research/investigators have identified differential expression of 25 miRs, including those that control embryonic stem cells transition to neuronal precursors (miR-106b, miR-21, miR-25, miR-30e, miR-598, miR-93)^[128]^. However, miRs, including miR-25, miR-17, miR-18, miR-19, miR-20a, miR-143, and miR-27, are repressed during neuronal lineage differentiation of somatic stem cells. Although most of the miRs are validated transcriptional targets of N-MYC, most are also known to target p53 and its downstream targets, and correlate with poor prognosis in NB^[128]^. While miR-181c is designated as a tumor suppressor, miR-181a/b is recognized as an oncomiR. Expression of miR181a/b was positively associated with N-MYC amplification and NB progression. It has been shown that miR-181a/b directly affects ABl1 expression and thereby dictates NB disease evolution^[129]^. Also, miR-380-3p has been recognized as an oncomiR, as it has been shown to promote tumor growth by repressing p53, and is associated with poor outcome in N-MYC-amplified NB^[130]^. Consistent with these observations, miR profiling studies clearly recognized that differential regulation of specific subsets of miRs could be beneficial for risk stratification in NB^[131]^.

## ROLE OF MIRS IN NB THERAPY RESISTANCE

NB-focused investigations indicated unique patterns of mutation(s) and specific miR dysregulation that could lead to therapy resistance^[66,68]^. In-depth analysis and crisscross comparisons of the documented functional roles of miRs across NB biological processes, including genesis, progression, therapy resistance, and evolution, clearly portrayed: (1) NB process-specific miR(s) regulation/deregulation; (2) within a process, function-specific miR(s) modulation; (3) that activation of function-specific miRs is complemented with compromised incompatible miR(s) (e.g., oncomiR activation with concomitant decrease of tumor suppressor miR or vice versa); and (4) the involvement of select miRs (e.g., miR-17-5p) across the function within a process and also across the processes of NB evolution [Figure 1, Table 1]. In particular, the miRs that dictate NB pathogenesis and tumor progression overlap to a large extent with those that orchestrate therapy resistance or sensitization. Differential modulation of at least seven miRs was reported in N-MYC-amplified NB cell lines (*vs.* N-MYC non-amplified) and further defined their role in orchestrated chromosomal imbalances^[68]^. A study in which primary NB tumors were screened revealed the underexpression of miRs in tumors with N-MYC amplification and demonstrated a substantial reversal with retinoic acid (RA) treatment, a compound that is known to reduce N-MYC expression and induce NB cell differentiation^[67]^. This study also showed that targeting N-MYC restores expression of miRs, indicating that N-MYC may mediate therapy resistance, in part, through directly or indirectly regulating the expression of miRs that are involved in cell differentiation, hence designating these miRs as potential therapeutic targets^[67]^.

In addition to the induced therapy resistance through well-directed underexpression of miRs that play a role in apoptosis and differentiation, studies have also shown that the function of miRs also heavily contribute to therapy resistance. Researchers have demonstrated that miR-21 could target PTEN and thereby increase NB cell proliferation and endorse chemo-resistance^[132]^. miR-21 promoter contains highly conserved regions with consensus binding sites for transcriptional regulator AP1 and forkhead family protein FOXO3a. Findings from one study indicated high expression of miR-21 in cisplatin-resistant NB cells, and its selective silencing prompted chemo-sensitization^[132]^. Interestingly, miR-21, the designated oncomiR, has been shown to be strongly upregulated under N-MYC knockdown conditions, and did not prompt any significant differentiation or proliferation with forced repression, indicating the probable requirement for miR-21 and N-MYC parallel activation for a causal effect^[133]^.

Five miRs within the polycistronic cluster (miR-17-5p, miR-18a, miR-19a, miR-20a, miR-92) were greatly expressed in NB cells with high N-MYC expression^[134]^. N-MYC binds directly to several sites of the 5’ and 3’ regions of this cluster candidate and hence could contribute to disease progression and therapy resistance^[134]^. While ectopic expression of miR-17-5p-92 has been shown to dramatically induce NB cell proliferation, miR-17-5p directly downregulates the tumor suppressor gene P21 by binding to its 3’ UTR region. Although cluster candidates miR-19 and miR-92 are known to modulate levels of the apoptotic BIM gene, it is now clear that miR-17-5p also targets BIM in NB cells. Targeting miR-17-5p with antagomiR profoundly inhibited NB growth *in vivo* and produced a reciprocal increase of both P21 and BIM^[134]^. In this regard, stabilizing miRs could serve as an all-inclusive strategy to overcome MDR, particularly for NB, since numerous miRs (miR-21, -128, -380-5P, -558, -375, -9, -15, -103, -107, -124, -29A, -152, -125b, -204, -363, -335, -17-92, and let 7) deregulate a panel of gene targets (discussed elsewhere in detail^[135]^) that orchestrate MDR.

It has been shown that the N-MYC-regulated miR-17-92 cluster inhibits NB cell differentiation through ERα repression, targeting nuclear hormone receptors, and suppression of glucocorticoid receptor expression associated with undifferentiated phenotype and decreased survival^[136]^. Chen and colleagues recognized an inverse expressional relationship between miR-137 and Constitute Androstane Receptor (CAR), a xenosensor and a key regulator of MDR^[137]^. This study showed that miR-137 is selectively downregulated in doxorubicin (Dox) -resistant NB cells; the stabilization (reinforcement) of miR-137 resulted in CAR protein suppression (effected through mRNA degradation) and promoted chemotherapy (Dox) sensitization. More importantly, the findings indicated the presence of a mechanistic negative feedback loop between miR-137 and CAR. Functionally, promoter hypermethylation and negative regulation of miR-137 by CAR contribute to the reduced miR-137 availability and increased CAR and MDR1, which contribute to Dox resistance^[137]^.

Loss of tumor suppressor miR-34a in progressive NB also contributes to therapy resistance. N-MYC is the direct target of miR-34a, with two functional target sites in the N-MYC 3’ UTR^[100]^. In addition to targeting N-MYC, mir-34a is known to target other crucial players of therapy resistance, including E2F3, BCl2, CCND1, and CDK6^[138]^. NB-targeted delivery of miR-34a not only decreased tumor growth, but also significantly reduced tumor vascularization by targeting TIMP2, a critical component of MDR^[139]^. In a similar study, Domenicotti and colleagues^[140]^. reported the function of miR-15a/16-1 in acquired drug resistance for NB. Interestingly, this study recognized a mono-allelic deletion of the 13q14.3 locus in p53, the location of p53-inducible miR15a/16-1 in etoposide-resistant cells. Acquired loss of miR-15a/16-1 leads to induced expression of BMI1 oncoprotein and activation of GSH-dependent responses, leading to etoposide resistance^[140]^. Ryan and colleagues^[141]^ reported that reinforcing the acquired loss of miR-204 targeted BCl2 and NTRK2, and thereby increased the sensitivity of NB cells to cisplatin. miR-204 is a tumor suppressor miR and is a designated predictor of outcome in NB.

Understanding how chemotherapy drugs inhibit cancer growth would place us in a better position to assess and/or prevent acquired resistance. Brain-derived neurotropic factor (BDNF) is a crucial protein that prompts neuronal cell survival, differentiation, and axon wiring through its interaction with tyrosine kinase receptor B (TrkB)^[142]^. BDNF-TrkB signaling has also been shown to affect the development, invasion, and outcome of many human tumors, including lung, bladder, pancreatic, and breast cancer. It has been shown that BDNF is the direct target of miR-61, and cisplatin treatment could significantly reinforce miR-61 and thereby inhibit BDNF in NB cells^[143]^. Similarly, miR-497 loss is associated with worse EFS and OS in N-MYC-amplified high-risk NB patients, and also corresponds with increased cell viability and decreased cell death. It has been shown that miR-497 directly targets WEE1, a key cell cycle regulator that orchestrates drug resistance. More importantly, ectopic expression of miR-497 or the inhibition of WEE1 resulted in significant chemotherapy sensitization to cisplatin, affirming that acquired loss of miR-497-dependent increased expression of WEE1 dictates drug resistance in NB cells^[144]^. Another study indicated a reduced expression of the tumor suppressor miR-376c-3p in the majority of the cell lines isolated from patients after IMCT^[145]^. miR-376c-3p directly targets Cyclin D1, an oncogene critical for NB pathogenesis. The therapy-associated acquired loss of miR-376c-3p in surviving NB cells illustrates its function in therapy resistance.

**Figure 2.**
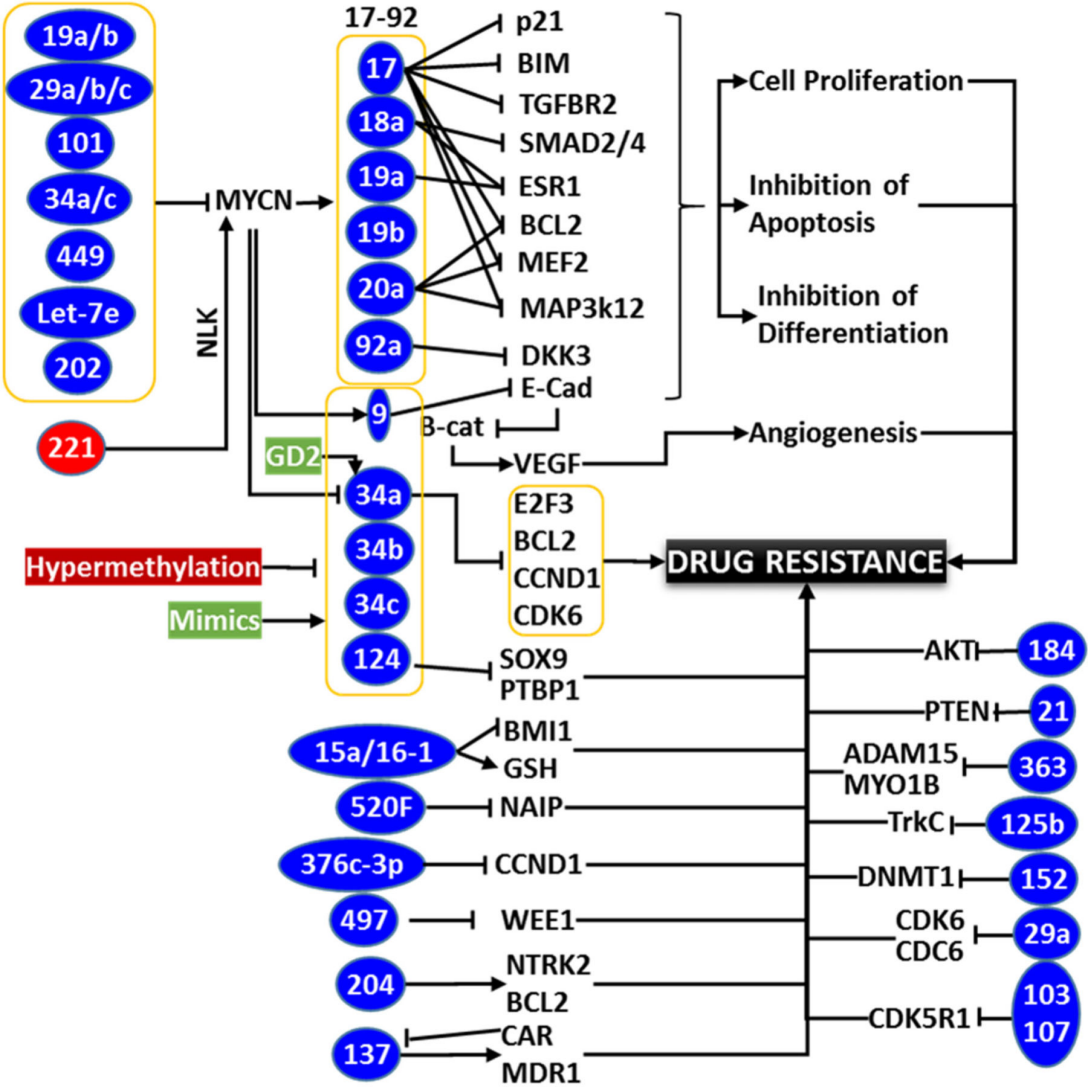
Schema showing the partial list of miRs involved in neuroblastoma (NB) therapy resistance. While MYCN regulated miRs have been documented to play crucial role in the therapy resistance through orchestrated clonal expansion and defying differentiation, acquired modulation of upstream miRs those regulate MYCN also plays crucial role in coordinating drug resistance and disease evolution. Hypermethylation of tumor suppressor miRs and hypomethylation of oncomiRs with clinical therapy in surviving cancer cells is regarded as one of the major mechanism for acquired loss of TS miRs and gain of oncomiRs, those dictate drug-resistance. Rearrangements on the levels of many key miRs inflicting therapy resistance through unique signaling flow-through are documented. Conversely, regulation/deregulation of many miRs converge on a signaling or functional event (e.g., Bcl2) to effect resistance. It is clearly evident that miRs play definitive roles in therapy resistance and miR-targeted approach could be an effective strategy for the treatment of resistant NB

In agreement with the hypothesis that both oncomiRs *vs.* tumor suppressor miRs drives therapy resistance, a high-throughput profiling study comparing miR deregulation across three independent chemo-resistant models consistently identified upregulation of four miRs (miR-150, miR-188-5p, miR-501-5p, miR-519B-3p) and downregulation of another three miRs (miR-125b-1, miR-204, miR-769-3p)^[146]^. Such emerging roles of miRs in exacerbating MDR highlight its criticality in clinical management of drug-resistant NB. Some of the key players involved in NB therapy resistance and their functional targets, the modulation of those that dictate therapy response are provided in Figure 2. One of the prime strategies in this setting is to block upregulated oncomiR by using antisense oligonucleotides. As identified earlier, targeted delivery of miR-34a using GD2-coated nanoparticles showed significant reduction in tumor growth, increased apoptosis, and reduced vascularization^[139]^. Conversely, the other method is to reinforce and to stabilize the downregulated tumor suppressor miR(s) using mimics. For instance, independent studies reinforcing tumor suppressor miRs (e.g. miR-9, miR-27b, miR-34a, miR-145, miR-184, *etc*.) showed a profound benefit reducing NB progression, and clearly indicated that these miRs could serve as novel therapeutic targets^[90,98,103,107,130,147,148]^. Chemotherapy in combination with miR-based treatment is currently recognized as a better strategy for clinical management of progressive NB. Ray and colleagues^[149]^ indicated that synthetic retinoid (4HPR) in combination with EGCG resulted in significant inhibition of oncomiRs miR-92, miR-93, and 106b, and induced the expression of tumor suppressors miR-7-1, miR-34a, and miR-99a. These findings suggest that treatments targeting oncomiRs and/or reinforcing tumor suppressor miRs would be highly beneficial in reverting induced therapy resistance in progressive NB.

Although it has been well recognized that MYCN amplification dramatically influences MDR by regulating an array of functional targets, including the genes involved in drug reflux, studies are highly limited in understanding the role of miRs in other high-risk subtypes that lack N-MYC amplification. In a recent study of NB chemo-resistance (resistance to cisplatin and cross-resistant to etoposide), it was shown that chromosomal rearrangement dictated significant overexpression of neural apoptosis inhibitory protein (NAIP) in drug-resistant cells^[150]^. Remarkably, this study identified significant loss of miR-520f in post-chemotherapy tumors (*vs.* pre-chemotherapy) and causally linked the loss of miR-520f to the induced expression of NAIP, which mediates drug resistance in this setting^[150]^. In parallel, an interesting study by Fabbri and colleagues^[151]^ indicated that the paracrine exchange of exosomic miRs between NB cells and neighboring human monocytes could critically affect drug resistance. NB cells secreting exosomic miR-21 led to monocyte-dependent miR-155 upregulation in a TLR8- and NFkB-dependent manner. Expressed miR-155 directly targets telomerase inhibitor TERF1 and affects telomerase activity and telomere length. This study also asserted the exosomic transfer of miR-155 from surrounding tumor-associated macrophages^[151]^. Researchers have also reported that exosomes carry as cargo various miRs, including miR-16, miR-125b, miR-21, miR-23a, miR-24, miR-25, miR-27b, miR-218, miR-320a, and miR-92a, which act as oncomiRs, affecting the sequence of many gene targets (e.g., NFkB, STAT3. P53, TLR8), and contributing to drug resistance^[152]^.

Due to the criticality of induced apoptosis or differentiation in NB cure, RA treatment is currently used as part of the NB treatment regimen^[153]^. Although RA treatment affects N-MYC regulation^[154]^ and other critical targets^[155]^ very early, it is generally accepted that these induced alterations could translate to differentiation or death. Since N-MYC directly regulates an array of miRs in NB, it is highly likely that RA treatment that inhibits N-MYC could inflict rearrangements. Assessing 70 miRs during RA-induced growth arrest identified upregulation of 14 miRs (miR-9, miR-124a, miR-125a, miR-125b, let-7a, let-7b, miR-7, miR-22, miR-23a, miR-24, miR-26a, miR-30a-5p, miR-100, miR-103) that are down-modulated in primary NB^[156]^. Functional analysis indicated the oncosuppressing potential of at least three miRs (miR-9, miR-125a, and b) and designated their possible use as diagnostic markers for tumorigenesis. An independent investigation by Stallings and Chen assessing the expression of 34 miRs (that exhibited significant variations between favorable *vs.* unfavorable NB) in NB cells exposed to RA treatment showed definitive upregulation of 17 miRs and downregulation of four miRs^[66]^. Interestingly, their outcomes indicated robust induction of miR-184, which functionally induced G1 cell cycle arrest, cell death, and complete suppression of miR181b and miR-92 (component of polycistronic cluster, highly expressed in N-MYC amplified NB^[134]^ and known to modulate apoptotic gene BIM)^[66]^. Independently, miR-184 has been shown to inhibit NB cell survival by directly targeting AKT and killing N-MYC-amplified cells^[101]^. Further, miR-10a and miR-10b have been causally linked to RA treatment-induced NB cell differentiation^[157]^. Of the 42 differentially expressed miRs in NB cells treated with RA, the authors indicated profound expression of miRs-10a and b and functionally characterized their role in RA-induced differentiation, RA-inhibited migration, invasion, and *in vivo* metastasis. Moreover, it has been recognized that miRs-10a and b directly target SR-family splicing factor (SRFS1), inhibit SRFS1-dependent alternative splicing and translational functions, and thereby orchestrate RA treatment-induced NB cell differentiation^[157]^. Another study indicated an up-modulation of miR-128, a brain-enriched miR, in RA-differentiated NB cells. Induced expression of miR-128 suppressed the expression of Reelin and DCX and reduced NB cell motility, invasiveness, and growth^[158]^. Reinforcing miR-128, which regulates the truncated isoform of NTRK3, has been shown to cause morphological changes in NB cells and alter the expression of genes involved in cytoskeletal organization, apoptosis, and cell proliferation^[159]^. miR-340 induces a cell-context-dependent differentiation or death, and has been shown to be upregulated through the demethylation process with RA-treatment^[69]^. Induced expression of miR-340 has been shown to inhibit Sox2, a critical stemness maintenance factor.

More importantly, a recent high-throughput NGS study comparing miR profiles in isogenic cell lines derived from NB patients at diagnosis and at relapse after IMCT identified 42 (8 upregulated and 34 downregulated) differentially expressed miRs^[160]^. Uniquely, 22 of 34 downregulated miRs were encoded from 14q32 miR cluster. Reduced expression of miRs from 14p32 cluster was significantly associated with poor prognosis in a cohort of 226 NB patients. Downstream gene target analysis of the roles of this cluster of miRs in signaling and biological functions recognized their association with disease progression and drug resistance^[160]^. Recent studies are now appropriately focused on elucidating the potential of targeted miR(s) delivery in the treatment of progressive tumors, including high-risk human NB^[161,162]^. Essand and colleagues demonstrated the benefit of miR-124, miR-125, and miR-134 insertion in SFV-4 virus in reducing the neuro-virulence and increased oncolytic capacity with an NB cure rate of about 50% in mouse model^[163]^. Similarly, it has been shown that enterovirus EV71 stimulates miR-Let-7b and directly targets CCND1 in therapy-resistant SH-SY5Y cells, identifying EV71 as a potential candidate for miR-based therapy in NB^[164]^. Another study indicated that fluoxetine, a serotonin reuptake inhibitor, upregulated miR-572 and miR-663a consistently in NB cells and thereby inhibited their crucial targets (mir-572 - Cdkn1, Dicer1, Wnt7a; miR-663a - TGFβ1, PTEN, VEGFA) associated with NB cell differentiation and tumor evolution^[165]^. The role of miRs in NB cell differentiation and the possibilities of targeting miRs for differentiation therapy is discussed in detail elsewhere^[166]^.

Novel strategies were adopted to investigate the benefit of delivering tumor suppressor miR-3245p, the independent predictor for NB^[167]^. Direct evidence demonstrating the benefit of a miR-based strategy for therapy-resistant NB was first sought by Fruci and colleagues. They showed that targeting miR-17-5p with antagomiR dramatically inhibited NB growth *in vivo* and showed a reciprocal increase of both P21 and BIM^[134]^. Later, an investigation by Li and colleagues recognized that miR-129 could inhibit tumor growth and, more importantly, promote NB cell sensitization to Cytoxan (chemo-drug often used in clinical treatment of NB) by selectively targeting MYO10^[168]^. Pal-Bhadra and colleagues identified novel conjugate [anthranilamide-pyrazolo (1,5-α) pyrimidine] -targeted miR-34-a, mir-34-c, mir-200b, mir-107, mir-542-5p, and mir-605, which led to the up-modulation of pro-apoptotic p21, Bax, and caspases, with concomitant downregulation of pro-survival Akt, E2F1, and Bcl2 in therapy-resistant human NB cells^[169]^. With the quantitative nuclear proteomics approach, studies have identified the mechanism of resveratrol, a natural phytoalexin-induced NB cell apoptosis. Resveratrol-induced miR-137 prompted the inhibition of polycomb protein histone methyltransferase EZH2, and reverted the EZH2-dependent regulation of tumor suppressors CLU and NGFR^[170]^.

With the evolution of the new concept that, “embryonic stem cell (ESC) gene regulatory mechanisms in tumors with high pluripotency capacity could contribute to the increased risk of therapy failure”, studies are now focused on understanding the realm of ESC miRs and their function in therapy resistance. Although this focus is still in its infancy, researchers are attempting to understand the basics. On that note, with an *in silico* approach, studies have listed a panel of ESC miRs along with mRNA signatures that are associated with poor NB patient outcomes, specifically for the N-MYC-amplified and N-MYC non-amplified high-risk subsets^[171]^. In addition, the findings indicated that the ESC signature is majorly driven by FOXM1 and could be useful to inhibit tumor progression and therapy resistance. Adding to the complexity of miR biology in NB disease evolution and therapy resistance, dose-associated differential modulation of miRs was also reported in NB cells. For instance, while low doses of morphine reduced the expression of miR-133b and prompted cell proliferation, higher doses inhibited cell proliferation, with reduced levels of miR-133b and miR-128^[172]^. In addition, new miRs residing within frequently altered chromosomal regions in NB tumors that could play significant functional roles in NB biology and therapy resistance are consistently being cloned^[173]^.

## CONCLUSION AND PERSPECTIVES

**Figure 3.**
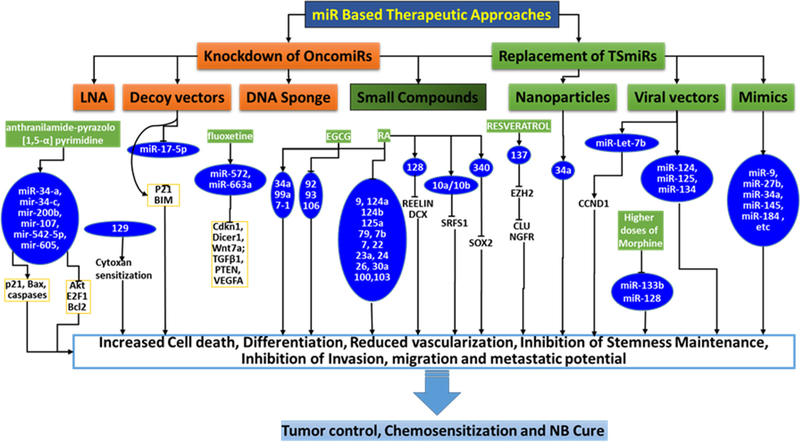
Schema showing the outline of the currently documented miR based treatment strategies for neuroblastoma (NB) cure. Independent studies validated various miR based strategies to increase cell death, inhibit clonal expansion, reduce vascularization, inhibit stem-like cell clonal selection and other crucial functions and, those effect NB cells for chemosensitization and NB cure

Despite tremendous clinical efforts with IMCT over the last two decades, a cure for high-risk NB is rare, with < 10% 5-year OS and < 2% long-term survival. These rates are mainly attributed to the continuous ongoing acquisition of genetic and molecular rearrangements in the NB cells that dictates therapy resistance and disease evolution. In the past decade, studies have been appropriately focused and many molecular targets have been proposed. Most of these proposed approaches have not reached clinical trials for multiple reasons, including that aiming single target or signaling in one of the NB evolution processes has been shown to result in activation of alternative and compensatory pathways, leading to additive resistance; cell-type-dependent expression and function; drug (treatment) type; and dose-dependent expression and function. Hence, it is essential to identify new and improved therapeutic strategies for high-risk NB, particularly through all-inclusive targeting that could counter acquired therapy resistance and disease progression. In this regard, miRs are an astonishing new class of gene regulators, and the discovery of the role of miRs in molecular pathogenesis of NB opened up their possible applications in NB diagnosis, prognosis, and miR-based therapy. The current wealth of information on contextual (diagnosis, prognosis, resistance) miR profiles; the direct roles of miRs in NB biology, cellular function, and signaling flow-through; and converging and/or compromising miR rearrangements recognizes that the miR-based therapy is around the corner. As discussed here, attempts were made and are also in progress to target oncomiR(s), reinforce tumor suppressor miR(s), or a combination [Figure 3]. Emerging evidence will further allow us to aim for (1) unique or converging molecular pathways; (2) specific cellular process; or (3) a combination of processes. Although translation of these preliminary research data into clinical application is in its infancy, these findings will provide a strong basis for translation and clinical use in the treatment of NB. In summary, we strongly opine that miRs that contribute to disease progression also have critical roles in the acquired molecular rearrangements and orchestrated therapy resistance of NB. Overall, the presented evidence supports the enormous clinical potential of miRs in countering therapy resistance and disease evolution, and warrants further rigorous investigation.

## Figures and Tables

**Table 1.** Partial list of microRNAs involved in NB disease progression and therapy resistance

miR	Type	HR-NB*	Target	Cellular function	NB process	Ref.
204	TSmiR	Inverse	N-MYC, BCL2, NTRK2	↑sensitivity to cisplatin	Chemo sensitization	[57]
2110	TSmiR	Inverse	TSKU	↑differentiation, ↑neurite	Oncosuppression	[61]
184	TSmiR	Inverse	AKT	↑cell death	Tumor suppression	[68,101]
17-5p	OncomiR	Direct	p21, BIM, ERα, NHR-GR	↑proliferation, ↓differentiation	Disease progression, therapy resistance	[134,136]
18a	OncomiR	Direct	p21, BIM, ERα, NHR-GR	↑proliferation, ↓differentiation	Disease progression, therapy resistance	[134,136]
19a	OncomiR	Direct	p21, BIM, ERα, NHR-GR	↑proliferation, ↓differentiation	Disease progression, therapy resistance	[134,136]
20a	OncomiR	Direct	p21, BIM, ERα, NHR-GR	↑proliferation, ↓differentiation	Disease progression, therapy resistance	[134,136]
92	OncomiR	Direct	p21, BIM, ERα, NHR-GR	↑proliferation, ↓differentiation	Disease progression, therapy resistance	[134,136]
221	OncomiR	Direct	NLK	↑cell cycle, ↑growth, ↑proliferation	↑n-MYC expression	[72]
34a	TSmiR	Inverse	MYCN, E2F3, BCL2, CCND1, CDK6, TIMP-2	↑cell cycle arrest, ↑apoptosis. ↓angiogenesis	Tumor suppression	[100,102,138,139]
9	TSmiR	Inverse	MMP14	↓invasion, ↓metastasis, ↓angiogenesis	Tumor suppression	[107]
340	TSmiR	↑ W/ RA	SOX2	↑differentiation, ↓stemness	Oncosuppression, chemosensitization	[69]
34b-3p	TSmiR	Inverse	CCNE2, E2F3	Regulates cell viability	Oncosuppression	[110]
203	TSmiR	Inverse	SAM68	↓proliferation, ↓invasion, ↓migration	Tumor suppression	[73]
337-3p	TSmiR	Inverse	MMP14	↓proliferation, ↓invasion, ↓migration Tumor suppression ↓angiogenesis		[74]
584-5p	TSmiR	Inverse	MMP14	↓proliferation, ↓invasion, ↓migration ↓angiogenesis	Tumor suppression	[75]
449a	TSmiR	Inverse	MFAP4, PKP4, TSEN15, CDK6, LEF1	↓growth, ↓survival, ↑cell cycle arrest ↑differentiation	Tumorigenesis, differentiation	[59]
506-3P	TSmiR	Inverse	CDK4 and STAT3	↑differentiation	Tumor suppression	[58]
124-3p	TSmiR	Inverse	CDK4 and STAT4	↑differentiation	Tumor suppression	[58]
193b	TSmiR	Inverse	MYCN, Cyclin D1, MCL1	↓viability, ↓proliferation, ↑cell cycle arrest, ↑cell death	Tumor suppression	[89]
145	TSmiR	Inverse	HIF 2α	↓growth, ↓metastasis, ↓angiogenesis	Tumor suppression	[90]
27b	TSmiR	Inverse	PPARγ	↓growth, ↓proliferation	Tumor suppression	[98]
542-3p	TSmiR	Inverse	Survivin	↓proliferation, ↑apoptosis	Tumor suppression	[99]
542-5p	TSmiR	Inverse	Survivin	proliferation, ↑apoptosis	Tumor suppression	[99]
335	TSmiR	Inverse	ROCK1, MAPK1, LRG1	↓invasion, ↓metastasis	Tumor suppression	[106]
210	TSmiR	Inverse	BCL2	↑apoptosis	Tumor suppression	[108]
181c	TSmiR	Inverse	MAD7	↓invasion, ↓metastasis, ↓angiogenesis	Tumor suppression	[109]
29a-3p	TSmiR	Inverse	CDK6, DNMT3A, DNMT3B	↓cell viability	Tumor suppression	[110]
517a -3p	TSmiR	Inverse	OLFM3, IFNAR1	Regulates cell viability	Tumor suppression	[110]
183	TSmiR	Inverse	85 targets, MCM 2-7	↓DNA replication	Tumor suppression	[112]
323a-5p	TSmiR	Inverse	CHAF1A, KIF11, E2F2, INCENP, CDC25A, CCND1, FADD	↓cell cycle arrest, ↑apoptosis	Tumor suppression	[116]
342-5p	TSmiR	Inverse	AKT2, CCND1, MKNK2, BCL-X	↓cell cycle arrest, ↑apoptosis	Tumor suppression	[116]
26A-5P	TSmiR	Inverse	LIN28B	↓oncogene	Oncosuppression	[117]
26B-5P	TSmiR	Inverse	LIN28B	↓oncogene	Oncosuppression	[117]
338-3p	TSmiR	Inverse	PREX2A	↓survival, ↓growth, ↑cell cycle arrest	Tumor suppression	[118]
1247	TSmiR	Inverse	ZNF346	↓proliferation, ↑cell-cycle arrest, ↑cell death	Tumor suppression	[121]
146a	TSmiR	Inverse	BCL11A	Inhibits cell growth and promotes apoptosis	Tumor suppression	[122]
558	OncomiR	Direct	HPSE, VEGF, AGO2, EIF4E	↑growth, ↑invasion, ↑metastasis, ↑angiogenesis	Tumor progression	[123,124]
451	OncomiR	Direct	MIF	↑growth, ↑invasion, ↑migration	Tumor progression	[125]
192	TSmiR	Inverse	Dicer1	↑proliferation, ↓migration	Tumor suppression	[126]
1303	OncomiR	Direct	GSK3β, SFRP1	↑ proliferation	Disease progression	[127]
181 a/b	OncomiR	Direct	ABL1	↑growth, ↑invasion	Disease progression	[129]
380-3p	OncomiR	Direct	p53	↑cell death	Disease progression	[130]
21	OncomiR	Direct	PTEN, APL, FOXO3A	↑ proliferation	Disease progression	[132]
137		↓CR cells	CAR	↑sensitivity to doxorubicin	Chemosensitization	[137]
15a/16-1		↓CR	BMI1	↓BMI1 and GSH	Chemosensitization	[140]
61	TSmiR	↑W/cisplatin	BDNF	↓survival, ↑differentiation	Chemosensitization	[142,143]
497	TSmiR	Inverse	WEE1	↓cell viability, ↑cell death	Tumor suppression	[144]
376c-3p	TSmiR	↓W/IMCT	Cyclin D1	↑cell death	Tumor suppression	[145]
155	TSmiR	Inverse	TERF1	↓telomerase activity, ↓telomere length	Tumor suppression	[151]
10A/10B	TSmiR	↑W/RA	SRFS1	↑differentiation, ↓migration, ↓invasion	Tumor suppression	[157]
128	TSmiR	↑W/RA	REELIN, DCX, NTRK3	↓motility, ↓invasion, ↓growth	Tumor suppression	[158,159]
129	TSmiR		MYO10	↓growth, ↑sensitization to Cytoxan	Chemosensitization	[168]

miR type (Tumor suppressor, TSmiR or OncomiR), their independent expressional association with high-risk disease (HR-NB*), gene targets that are directly targeted by miRs, cellular/biological functions, their role in NB disease evolution processes, and the studies that investigated their functions are listed. ↑: induces/increases; ↓: inhibits/reduces; W: with; RA: Retinoic acid treatment; CR: in chemoresistant cells; IMCT: intensive multimodal clinical therapy; NB: neuroblastoma
